# Emotional Tears Communicate Sadness but Not Excessive Emotions Without Other Contextual Knowledge

**DOI:** 10.3389/fpsyg.2019.00878

**Published:** 2019-04-24

**Authors:** Kenichi Ito, Chew Wei Ong, Ryo Kitada

**Affiliations:** Division of Psychology, School of Social Sciences, College of Humanities, Arts, and Social Sciences, Nanyang Technological University, Singapore, Singapore

**Keywords:** tear, face perception, facial expressions, emotion, intensity rating, multidimensional scaling

## Abstract

Contexts of face perception are diverse. They range from the social environment to body postures, from the expresser’s gaze direction to the tone of voice. In extending the research on contexts of face perception, we investigated people’s perception of tears on a face. The act of shedding tears is often perceived as an expression of sad feelings aroused by experiencing loss, disappointment, or helplessness. Alternatively, tears may also represent the excessive intensity of any emotion, such as extreme fear during an unexpected encounter with a giant bear and extreme happiness when you win a competition. Investigating these competing interpretations of tears, we found that the addition of tears to different facial expressions made the expressions conceptually closer to sad expressions. In particular, the results of the similarity analysis showed that, after the addition of tears, patterns of ratings for anger, fear, disgust, and neutral facial expressions became more similar to those for sadness expressions. The effect of tears on the ratings of basic emotions and their patterns in facial expressions are discussed.

## Introduction

Detecting and interpreting facial expressions of emotion are essential parts of human interaction. There are two main approaches to understanding how people interpret facial expressions. Researchers employing the basic emotion approach adopt the view that discrete and universal emotions could be identified through certain facial muscular movements independently (Ekman et al., 1982; Nakamura et al., 1990). Conversely, researchers taking a situational approach stress the importance of context in attributing meaning to facial expressions (Smith, 1989; Fernández-Dols et al., 1991; Carroll and Russell, 1996; Aviezer et al., 2012). Recent studies investigating the role of context have demonstrated the integration of information from contexts and facial expressions in emotion attribution. This integration of contextual cues occurs automatically, to the extent that it overpowers the emotional cues from facial expressions (Aviezer et al., 2011). These findings showed a significant impact of contexts in the perception of facial expressions and challenged the notion of universal, basic facial expressions (Wieser and Brosch, 2012; Aviezer et al., 2017).

Contexts in facial expressions can be grouped into several categories, including observer-based contexts, cultural contexts, and stimulus-based contexts (Barrett et al., 2011). One example of observer-based contexts is the difference in observers’ language and semantic knowledge. In particular, reducing observers’ access to emotion language through semantic satiation lowered the accuracy in judging distinct facial expressions (Lindquist et al., 2006). A well-known example of cultural contexts is the own race bias. That is, people identify facial expressions of others from the same or similar cultural backgrounds better than those from relatively distant cultural backgrounds (e.g., Elfenbein and Ambady, 2003; Elfenbein et al., 2007). Stimulus-based contexts consist of sensory input with emotional information on top of facial expressions. Some examples are congruent emotional sounds (Müller et al., 2011) that intensify facial expressions, body postures that bias the categorization of facial expressions (Meeren et al., 2005), and colors of a face that communicate expressers’ emotional states independently of facial muscle movement (Benitez-Quiroz et al., 2018).

Within the research on stimulus-based contexts, congruence between the signal value of the eyes and facial expressions is known to facilitate perception of facial expressions. For example, the direction of an expresser’s gaze helps a perceiver to identify the source of threat and foster the immediate and accurate perception of an emotion (Adams and Kleck, 2003, 2005). Specifically, a direct gaze that is congruent with the approach orientation of anger expressions and happy expressions was found to enhance the perception of both anger and happiness. In contrast, an averted gaze that is congruent with the avoidance orientation of fearful expressions and sadness expressions was found to enhance the perception of both fear and sadness. These findings in combination supported the notion that gaze, as a stimulus-based context, facilitates perception of facial expressions when the signal values of both gaze and facial expressions are congruent. In addition to gaze, reddening of the sclera (i.e., the white outer layer of the eye), another stimulus-based context, was found to intensify the perception of angry, disgusted, fearful, and sad expressions while reducing the intensity of happy expressions (Provine et al., 2013). These examples advanced our understanding of contextual influences by suggesting a significant effect of stimulus-based contexts on the perception of facial expressions. Besides eye gaze and reddened sclera, recent studies have investigated tears as stimulus-based contexts.

A tear rolling down one’s cheek in response to his or her feeling and thinking is called an emotional tear (Murube, 2009). Other than emotional and cognitive reasons, tears could be produced to nourish and lubricate an ocular surface (i.e., basal tears) or to wash away dust or other unwanted substances from eyes (i.e., reflex tears), as seen in behaviors such as yawning, coughing, and vomiting. However, a crying person is often interpreted as experiencing turbulent feelings and thoughts. Such an interpretation makes an emotional tear a stimulus-based context that modulates the perceived intensity of facial expressions, and interpreting tears might involve both automatic and controlled processes similar to perceiving emotions from facial expressions (Adolphs, 2002; Balsters et al., 2013). Additionally, it is proposed that emotional tearing, a unique human behavior, serves to communicate information to observers in order to change their behavior in a way that benefits the tearing individual (Gračanin et al., 2017). For instance, emotional tearing could signal appeasement or need for attachment, which in turn fosters social bonding (Hasson, 2009). Thus, it is not surprising that tears could communicate emotional information in accordance with the aforementioned social signals.

An increasing number of studies have examined emotional tears as stimulus-based contexts in emotion perception (e.g., Vingerhoets and Cornelius, 2001; Hendriks and Vingerhoets, 2006; Provine et al., 2009; Balsters et al., 2013; Reed et al., 2015; Takahashi et al., 2015; Vingerhoets et al., 2016; van de Ven et al., 2017; Küster, 2018). For example, Provine et al. (2009) digitally removed tears from images of natural tearful faces, and they found that participants rated the images with tears as sadder than the images without tears. Also, this finding was consistent across observers of both genders and of different ages. Thus, the authors proposed a sadness-enhancing effect as a result of tears in emotion attribution. In another study, the sadness-enhancing effect of tears was found at a pre-attentive level (Balsters et al., 2013). Images of neutral, sad, and tearful-sad expressions were presented for a very brief duration (i.e., 50 ms), and observers identified tearful-sad expressions the fastest among other expressions. Moreover, observers reported that they were unaware of the presence of tears in the images, showing that tears could affect sadness perception even without observers’ conscious awareness. Takahashi et al. (2015) found that tears increased sadness of facial expressions, as compared to expressions with gray circles resembling tears and expressions without tears. Moreover, the effect of tears was stronger for neutral expression than for sad expressions, indicating that the effect of tears is stronger for non-sad facial expressions. Lastly, the effect of tears on sadness perception differed with the expressers’ age groups, with the effect being most pronounced when the expressers were adults, followed by children and infants (Zeifman and Brown, 2011). This suggested that tears gain significance as visual signals of sadness over the course of life.

In contrast to the sadness enhancement hypothesis, which suggests that emotional tears primarily increase the perceived intensity of sadness, emotional tears may increase the intensity of the primary emotion expressed by each facial expression. That is, the effect of emotional tears may not be limited to sadness. Rather, emotional tears are perceived as products of emotions that have passed a critical threshold of stress or tension (Vingerhoets and Cornelius, 2001). From this general enhancement perspective, the act of shedding tears is a coping mechanism to avoid excessive accumulation of not only sadness but also other emotions. Individuals maintain homeostasis of mind and body by releasing emotions in the form of tears (Frijda, 1986; Vingerhoets and Cornelius, 2001). The general enhancement hypothesis posits that emotional tears amplify the intensity of facial expressions, and thus, perceivers are expected to report greater intensity of emotions congruent with an expresser’s facial expressions. For instance, an angry expression with tears would be perceived as angrier.

Reed et al. (2015) conducted a study in which participants observed and rated video clips of crying individuals who posed universal facial expressions of sadness, fear, anger, happiness, and neutrality based on the Facial Action Coding System (FACS; Ekman and Friesen, 1978). The results were mixed. On the one hand, participants showed a tendency consistent with the sadness enhancement hypothesis in that they reported a higher intensity of perceived sadness in tearful individuals than non-tearful individuals across different facial expressions. On the other hand, participants showed a tendency consistent with the general enhancement hypothesis in that perceived intensity of anger was significantly higher for angry expressions with tears than angry expressions without tears, and perceived intensity of fear was marginally higher for fearful expressions with tears than fearful expressions without tears. Thus, it remains unclear which hypothesis explains the effect of tears on facial expressions more strongly than the other.

One approach to addressing this issue is to use representational similarity analysis (RSA, Kriegeskorte et al., 2008) and multidimensional scaling (MDS) to visualize the effect of tears on facial expressions (Adolphs et al., 1994; Kennedy and Adolphs, 2012). For instance, each facial expression can be mapped in the dimensions of basic emotions when each stimulus was evaluated in terms of the intensity of these basic emotions. As correlations of ratings between stimuli (*r*) indicate similarity between stimuli, 1 – *r* indicates dissimilarity between stimuli. The MDS creates a map of stimuli in Cartesian space with reduced dimensions wherein the distance between stimuli represents inter-stimulus dissimilarity. Locations of emotional facial expressions within the MDS space were similar to those of emotions Russell’s circumplex model (Russell, 1980) and have been used to characterize the emotion perception of patients with damage to the bilateral amygdala (Adolphs et al., 1994) and autistic individuals (Kennedy and Adolphs, 2012).

In the present study, we used RSA and MDS to examine the effect of tears on the perceived intensity of basic emotions of facial expressions, using neutral and negative facial expressions (i.e., anger, disgust, fear, and sadness). That is, we investigated whether the addition of tears will change the intensity of perceived sadness, anger, disgust, and fear. We focused on negative facial expressions because previous research reviewed above showed support for both hypotheses especially when perceivers responded to negative facial expressions. If the sadness enhancement hypothesis is supported, sadness ratings of all facial expressions would increase, and the ratings of other emotions of all facial expressions would remain the same or decrease. Because the rating patterns of facial expressions become similar to those of sad expressions, the distances between sad expressions and other facial expressions in MDS space (calculated based on 1 – *r*) will be reduced (Supplementary Table S1). By contrast, if the general enhancement hypothesis is supported, tears would increase the ratings of the emotion that is primarily portrayed by each facial expression; for instance, tears would increase the intensity of anger but not the intensity of other emotions in an angry expression. In this case, because the increase in ratings in different dimensions keeps the correlations the same (Supplementary Table S1), the distances (dissimilarities) in MDS space will remain the same.^1^

## Materials and Methods

### Participants

Participants were 98 Americans who were recruited through Amazon MTurk (53.1% males; 80.6% Caucasian Americans; *M*_age_ = 34.45 years, *SD* = 10.29). We referred to previous studies (Provine et al., 2009; Reed et al., 2015) to determine the sample size for our study. Also, our sample size exceeded the required sample size of 24, estimated using a power analysis with the effect size of.26, which we obtained from Provine et al. (2009) study, alpha of 0.05, and beta of 0.95.

### Stimuli

**FIGURE 1:**
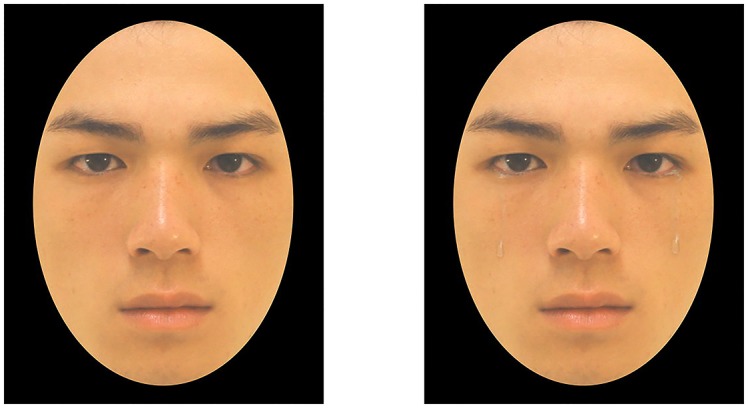
An example of a neutral facial expression without tears (left) and the same image with digitally added tears (right). Images are from Chen and Yen (2007). *Taiwanese Facial Expression Image Database*. Brain Mapping Laboratory, Institute of Brain Science, National Yang-Ming University, Taipei, Taiwan. No further permission was required for the use of these images.

We used standardized images of Asian faces showing neutral, fearful, sad, disgusted, or angry expressions with a resolution of 480 × 640 pixels (Chen and Yen, 2007). All images were cropped to exclude hair, ears, and other external features. In selecting the stimuli, we had 20 MTurk participants categorize 28 models who expressed seven facial expressions (neutral, fear, sadness, disgust, anger, happiness, or surprise) in random order. The participants in the pretest did not participate in the main study. Overall, participants’ accuracy in categorizing all seven facial expressions for each model ranged from 71.43% to 95.71%. We selected six models (three females) whose facial expressions were accurately categorized at least 80% of the time. We then digitally added tears to the facial expressions. Tears were placed near the nostrils, as shown in Figure 1. The final set consisted of 60 images. By using the same models to pose for different facial expressions, we avoided confounding the effect of tears with models’ facial characteristics.

### Procedure

Participants were randomly assigned to either the tearful or the non-tearful condition. In each trial, participants viewed a facial expression and rated the intensity of anger, disgust, sadness, and fear from 0 (*not at all)* to 100 (*very strong*) using four visual analogue scales. We followed past studies (Adolphs et al., 1994; Provine et al., 2009; Reed et al., 2015) in using intensity ratings for each expression, because intensity ratings may detect the subtle effect of tears. The visual analogue scale is an appropriate method to obtain continuous data and was used by Takahashi et al. (2015) for the perceived intensity of sadness. There was no time limit for participants to respond to each trial. The protocol and consenting procedure of this study were approved by Nanyang Technological University’s Institutional Review Board, IRB-2017-05-016, entitled “Psychological impact of tearful face on social judgment.”

### Statistical Analyses

**FIGURE 2:**
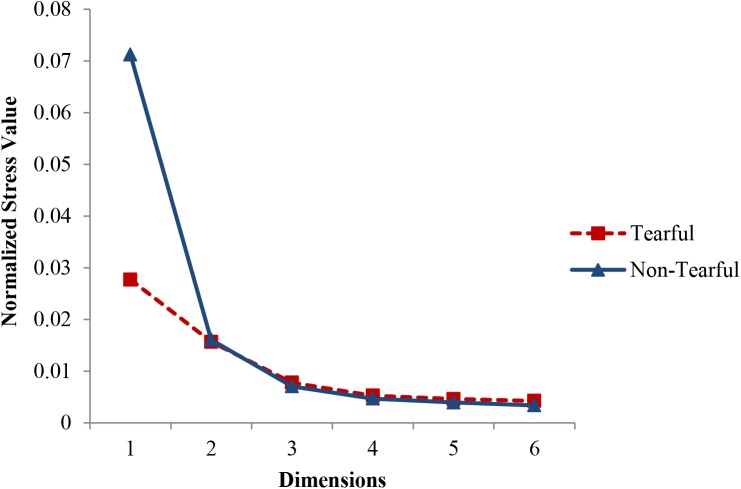
Scree plots for normalized stress values in MDS for tearful condition (*n* = 50) and non-tearful condition (*n* = 48).

In the present study, we obtained similarities among stimuli using a method accepted in neuroscience (see Kriegeskorte et al., 2008, for a description of RSA). More specifically, we measured correlations of ratings between any pair of stimuli and treated them as similarities. Although the conventional methodology in psychology utilizes ratings of similarity between stimuli, correlations of ratings can be utilized to visualize such similarities or dissimilarities (Groenen and Borg, 2013). Using the same method as the current study, Adolphs et al. (1994) and Kennedy and Adolphs (2012) successfully visualized typical and atypical representational spaces of facial expressions. We used non-metric MDS to visually examine the data and test the hypotheses. In the current study, we derived similarities by correlating each participant’s intensity ratings of one facial expression with those of every other facial expression. The correlation coefficients in the individual similarity matrices were transformed using Fisher’s r-to-z transformation. Then, the similarity matrices based on transformed values for tearful (*n* = 50) and non-tearful (*n* = 48) conditions were averaged and inverse-transformed back into Pearson’s r (see Kennedy and Adolphs, 2012, for a similar method). Dissimilarity indices were calculated with 1 – *r* so that a larger number indicates a larger perceived dissimilarity between two facial expressions. The dissimilarity indices were then analyzed with the PROXSCAL program in SPSS. Scree plots showing stress values for one- to six-dimensional solutions in both the tearful and non-tearful conditions (Figure 2) indicated that the drop in stress value (or the elbow) was substantial at two dimensions and minimal from three dimensions onward. Stress values for a two-dimensional solution were good (stress = 0.016 for both tearful and non-tearful conditions; Clarke, 1993). Therefore, we selected a two-dimensional solution. Previous studies on facial expressions using a similar method of analysis also deemed a two-dimensional solution appropriate (Adolphs et al., 1994; Kennedy and Adolphs, 2012). Based on each participant’s MDS solution, we obtained his or her unique coordinates for the center of each facial expression. Then, we calculated the Euclidean distance between the center of sad expressions and the center of angry, fearful, disgusted, or neutral expressions to test the hypotheses. Data are available on https://doi.org/10.21979/N9/SQCDCB.

## Results

We performed a 2 (tears: tearful vs. non-tearful) × 5 (model emotions: neutral, sadness, anger, disgust, fear) × 4 (emotion categories for rating: sadness, anger, disgust, fear) × 2 (model biological sex: men vs. women) × 2 (participant biological sex: men vs. women) analysis of variance (ANOVA) with tears and participant biological sex being between-subject variables and model emotions, emotion categories for rating, and model biological sex being within-subject variables, and participants’ intensity rating for each expression being a dependent variable. The assumption of sphericity was not met; thus, Greenhouse–Geisser correction was used for analysis involving the two within-subject factors. We included model biological sex and participant biological sex to examine a possible sex effect on intensity ratings. However, the main effects of model biological sex and participant biological sex and the interaction effect involving model biological sex × participant biological sex were not significant, *Fs* < 1.33, *ps* > 0.19. Thus, we will not discuss this issue further for the sake of brevity.

**FIGURE 3:**
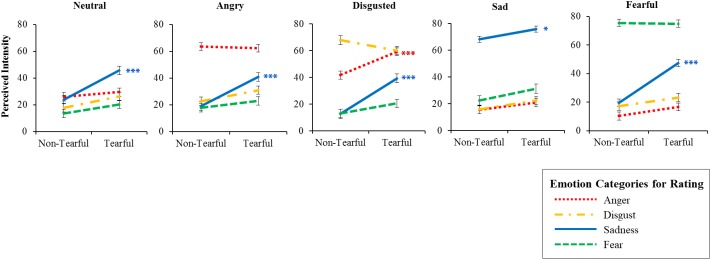
Intensity ratings for tearful and non-tearful expressions. Error bars represent the standard error of the mean. ^∗^*p* < 0.05, ^∗∗∗^*p* < 0.001.

**FIGURE 4:**
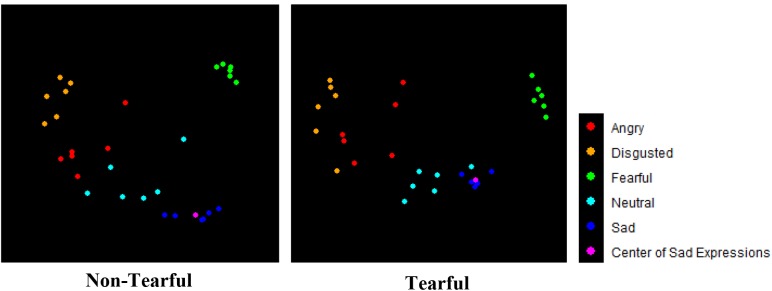
Multidimensional scaling plots for dissimilarity among different facial expressions for tearful condition (*n* = 50) and non-tearful condition (*n* = 48). Dots represent stimuli, the colors represent facial expressions, and the distances between dots represent the dissimilarities.

The analysis revealed significant main effects of tears, *F*(1,94) = 7.347, *p* = 0.008, $\eta_{p}^{2}$ = 0.072, model emotions, *F*(3.000,282.022) = 81.148, *p* < 0.001, $\eta_{p}^{2}$ = 0.463, and emotion categories for rating, *F*(2.295,215.734) = 21.803, *p* < 0.001, $\eta_{p}^{2}$ = 0.188. More importantly, we found a significant interaction effect of tears × model emotions × emotion categories for rating, *F*(3.634,341.605) = 4.955, *p* = 0.001, $\eta_{p}^{2}$ = 0.050. As shown in Figure 3, simple effect analysis showed that participants reported greater intensity of sadness for tearful condition than non-tearful condition for angry expressions, *F*(1,94) = 21.324, *p* < 0.001, $\eta_{p}^{2}$ = 0.185, *M*_difference_ = 20.319, 95% CI [11.583, 29.056], disgusted expressions, *F*(1,94) = 28.577, *p* < 0.001, $\eta_{p}^{2}$ = 0.233, *M*_difference_ = 25.169, 95% CI [15.820, 34.517], fearful expressions, *F*(1,94) = 29.539, *p* < 0.001, $\eta_{p}^{2}$ = 0.239, *M*_difference_ = 2.877, 95% CI [17.058, 36.696], neutral expressions, *F*(1,94) = 23.422, *p* < 0.001, $\eta_{p}^{2}$ = 0.199, *M*_difference_ = 21.509, 95% CI [12.685, 30.333], and sad expressions, *F*(1,94) = 7.362, *p* = 0.008, $\eta_{p}^{2}$ = 0.073, *M*_difference_ = 8.641, 95% CI [2.318, 14.94], supporting the sadness enhancement hypothesis. In addition, the intensity of anger for disgusted expressions was higher for tearful condition than non-tearful condition, *F*(1,94) = 16.824, *p* < 0.001, $\eta_{p}^{2}$ = 0.152, *M*_difference_ = 16.795, 95% CI [8.665, 24.925]. This finding will be discussed in the general discussion section.

Subsequently, we performed MDS to visually examine the similarities of different facial expressions in tearful and non-tearful condition, respectively. Two types of MDS analysis were conducted. First, we conducted MDS with the group dissimilarity matrices (one for tearful condition and another for non-tearful condition). Figure 4 shows the dissimilarity of intensity ratings for facial expressions with or without tears in the group data. The averaged distance between each sad expression and every other facial expression in MDS showed that facial expressions with tears were more clustered toward sad expressions (or less dissimilar) than those without tears (distance*_tearful_* = 0.877, distance*_non-tearful_* = 1.073), supporting the sadness enhancement hypothesis. Highly similar patterns were observed even when we conducted the same analysis by including facial expressions with and without tears together in one MDS space (Supplementary Figure S1 and Supplementary Table S2).

Second, we generated individual dissimilarity matrix and conducted individual MDS for each participant to allow us to use inferential statistics. We tested the sadness enhancement hypothesis by calculating the Euclidean distances between the mean point of sad expressions (indicated as center of sad expressions in Figure 4) and those of the other four expressions, which indicate the dissimilarity between participants’ intensity ratings of sad expressions and other expressions. A 2 (tears: tearful vs. non-tearful) × 4 (model emotions: neutral, angry, disgusted, fearful) ANOVA with the Euclidean distances between center of sad expressions and the other four expressions as dependent variable showed significant main effects of model emotions, *F*(2.703,259.502) = 52.812, *p* < 0.001, $\eta_{p}^{2}$ = 0.355 and tears, *F*(1,96) = 17.802, *p* < 0.001, $\eta_{p}^{2}$ = 0.156. Tears × model emotions interaction was not significant, *F*(2.703,259.502) = 0.132, *p* = 0.927, $\eta_{p}^{2}$ = 0.001. Supporting the sadness enhancement hypothesis (see Table 1), the Euclidean distances from the center of sad expressions were significantly shorter in the tearful condition as compared to the non-tearful condition for angry expressions, *F*(1,96) = 10.637, *p* = 0.002, $\eta_{p}^{2}$ = 0.100, *M*_difference_ = 0.179, 95% CI [0.070, 0.288], disgusted expressions, *F*(1,96) = 4.553, *p* = 0.035, $\eta_{p}^{2}$ = 0.045, *M*_difference_ = 0.138, 95% CI [0.010, 0.266], fearful expressions, *F*(1,96) = 8.312, *p* = 0.005, $\eta_{p}^{2}$ = 0.080, *M*_difference_ = 0.153, 95% CI [0.048, 0.259], and neutral expressions, *F*(1,96) = 7.946, *p* = 0.006, $\eta_{p}^{2}$ = 0.076, *M*_difference_ = 0.145, 95% CI [0.043, 0.247].

**Table 1:** Mean Euclidean distances from center of sad expressions.

	Tearful	Non-tearful	*F*
Anger	0.71 *(0.32)*	0.88 *(0.21)*	10.637**
Disgust	0.90 *(0.34)*	1.04 *(0.30)*	4.553*
Fear	0.66 *(0.27)*	0.81 *(0.26)*	8.312**
Neutral	0.46 *(0.20)*	0.61 *(0.30)*	7.946**


*Standard deviations are given in parentheses. ^∗^*p* < 0.05; ^∗∗^*p* < 0.01.*

Finally, we conducted two supplemental analyses to confirm the result above. First, we compared the dissimilarity measure (1 – *r*) before the MDS transformation. The group mean of the dissimilarities between sad expressions and other facial expressions was 0.69 for tearful expressions and 0.97 for non-tearful expressions. The difference in dissimilarity (-0.28) is large enough to support the sadness enhancement hypothesis, even if the assumption of emotion ratings in the general enhancement hypothesis (Supplementary Table S1) are altered (Supplementary Figure S2). In the second analysis, we used different similarity/dissimilarity measures to complement the first analysis. That is, we mapped the stimuli in a four-dimensional Cartesian space, each of which indicates the intensity rating of each emotion (anger, disgust, fear, and sadness). We calculated the Euclidean distances between sad expressions and other facial expressions. In this distance measure, the sadness enhancement hypothesis predicts that tears will either reduce the distances or will not change them, whereas the general enhancement hypothesis predicts that tears will increase the distances (see Supplementary Figures S2, S3 and Supplementary Table S1). This supplemental results also showed that the distances from sad expressions were shorter for tearful facial expressions than non-tearful facial expressions (Supplementary Table S3). Finally, the two-way ANOVA [(two tears: tearful vs. non-tearful) × (four model emotions: anger, disgust, fear, neutral)] on the distances showed significant main effects of tears, *F*(1,96) = 5.794, *p* = 0.018, $\eta_{p}^{2}$ = 0.057, and model emotions, *F*(2.443,234.483) = 69.983, *p* < 0.001, $\eta_{p}^{2}$ = 0.422. The interaction effect of tears × model emotions was not significant, *F*(2.443,234.483) = 1.436, *p* = 0.238, $\eta_{p}^{2}$ = 0.015. This result confirms that in the supplemental analysis, tears reduced the distance (i.e., increased similarity) between sad expressions and other facial expressions.

## Discussion

The current study used RSA and MDS to address whether the effect of emotional tears on facial expressions of emotion is consistent with either the sadness enhancement hypothesis or the general enhancement hypothesis. Past studies showed that the presence of tears increased the perceived intensity of sadness across different facial expressions of emotion. However, a close examination of the results also supported the general enhancement hypothesis when angry expressions with tears were perceived to be angrier than the same angry expressions without tears. Because these past studies adopted univariate analyses on examining the tears effect (i.e., they only provided the statistical test on each emotion rating), it is unclear how strongly each hypothesis was supported by the data. Multivariate analysis such as RSA and MDS can complement such shortcomings by integrating all ratings as inter-stimulus dissimilarity and distance. Through testing these two hypotheses derived from distinct theoretical backgrounds, we extended the understanding of the effect of tears on facial expressions by demonstrating that the dissimilarity space among facial expressions tends to shrink toward sad expressions. In the present study, the overall pattern of the tears effect favors sadness enhancement hypothesis rather than general enhancement hypothesis.

We can assume several cases of sadness enhancement and general enhancement hypotheses (Supplementary Table S1). First, the sadness enhancement hypothesis could predict an increase in sadness ratings in sad expressions at a lesser extent than in the other expressions. This assumption is based on Weber’s law, which states that the change in stimulus intensity that can be discriminated (i.e., just noticeable difference) is a constant fraction of the intensity of the original stimulus. Indeed, previous study demonstrated that two highly intense expressions (e.g., 70% and 90% intensity of one emotion) are perceived as more similar than two less intense expressions with the same physical discrepancy (e.g., 50% and 70%; Gao et al., 2010, 2013). The effect of tears was smaller for sad expressions than neutral expressions (Takahashi et al., 2015). However, even if this assumption is not true, the sadness enhancement hypothesis still predicts results that are different from what the general enhancement hypothesis predicts: tears lead to reduced dissimilarities in the main analysis (1 -*r*) and unchanged dissimilarities in the supplemental analysis (Euclidean distance) (Supplementary Figure S2). Similarly, the general enhancement hypothesis could predict either a uniform increase in emotion ratings in all expressions or differences in such increments. However, regardless of the different predictions, the general enhancement hypothesis still predicts that tears lead to smaller changes in dissimilarities than the sadness enhancement hypothesis in the main analysis (1 -*r*) and increased dissimilarities in the supplemental analysis (Euclidean distance). Collectively, although detailed patterns of the tears effect may vary within each hypothesis, such differences do not affect our conclusion on which hypothesis our data support.

It is possible that our predicted changes in MDS space are not specifically tied to either the sadness enhancement hypothesis or general enhancement hypothesis, and it could be explained by other patterns of changes in the intensity rating of emotions. We discuss a few possibilities here. For instance, tears could increase sadness ratings of sad expressions but do not change any emotion ratings of other facial expressions. In this case, the distances between sad expressions and other facial expressions in MDS space would remain the same in the main analysis (or be increased in the supplementary analysis). Next, tears could reduce all emotion ratings in all facial expressions, and thus the distances between sad expressions and other facial expressions would remain the same (or be decreased in the supplementary analysis). Also, tears could increase all emotion ratings of all facial expressions, and thus the distances between sad expressions and other facial expressions would remain the same (in both main and supplementary analyses). However, these possibilities are unlikely, given that they are inconsistent with the result of univariate analyses that showed an increase in sadness ratings in all facial expressions (Figure 3).

Although we maintain that emotional tears primarily signal the expresser’s experience of sadness, the general enhancement hypothesis can explain people’s experience of shedding tears, especially when contexts warrant the expression of intense emotions. For example, when athletes shed tears after they won a gold medal, people perceive extreme happiness from these tears. When a victim of a robbery burst into tears, people perceive extreme fear from these tears. We speculate that the sadness enhancement hypothesis explains people’s perception of tears when contextual cues are ambiguous and moderate in intensity, while the general enhancement hypothesis can explain the perception of tears when contextual cues are clear and intense.

Interestingly, our study showed that disgusted expressions with tears were seen as angrier than disgusted expressions without tears. This pattern of results replicated Widen and Russell (2008) study, in which children and adults would misinterpret disgusted expressions as showing anger. However, they were less likely to misinterpret angry expressions as showing disgust, indicating that the misinterpretation was unique to disgusted expressions. This corresponded with our results showing that angry expressions with tears were not seen as more disgusted than angry expressions without tears. The additional emotional information introduced by tears could have led to greater uncertainty about the emotion expressed. This increased uncertainty brought about by tears might have then amplified any confusion in perceiving disgusted expressions. While our findings on disgusted expressions supported the sadness enhancement hypothesis (i.e., the presence of tears made the expressions look sadder instead of more disgusted), future studies could focus on how tears change the interpretation of vague facial expressions of emotion such as disgust.

We focused on examining the effect of tears on negative facial expressions in examining the two competing hypotheses as past research has shown mixed findings on the effect of tears on negative expressions. The presence of tears on happy expressions has been shown to either decrease or have no effect on perceived happiness (Reed et al., 2015). Given that tears increased the intensity of sadness in all facial expressions in the present study, it is not clear if the inclusion of happy expressions could change the effect of tears on inter-stimulus distance to the extent that the general enhancement hypothesis is favored. Nevertheless, it is important to examine the extent to which the inclusion of positive facial expressions influences the MDS space. Moreover, while past research did not support the general enhancement hypothesis in positive expressions, we are aware that in certain contexts people who are tearing could be seen as experiencing extreme positive emotions (e.g., the example earlier on tearing athletes being perceived as experiencing extreme happiness). Besides, tearing expressions, more often than not, occur with the presence of other contextual information. Thus, collectively, it is important in the future study to consider the inclusion of happy expressions and identify additional contextual factors that affect the interpretation of tearing expressions.

There are several methodological and interpretational issues. First, the main difference between our method and conventional methods is that our dissimilarity space is limited to dimensions of basic emotions used for ratings. This is because we did not include other information beyond basic facial expressions (e.g., facial identity). Future studies looking at whether our findings are applicable to dimensions beyond basic emotions are necessary.

Second, the MDS space in our study appears to be different from Russell (1980) MDS space in which the vertical axis represents arousal and the horizontal axis represents valence. The vertical axis in our study is likely to represent arousal level, whereas the horizontal axis is unlikely to represent valence, as neutral expressions are located between negative facial expression instead of at either end of the horizontal axis that would suggest changes in negative valence. This difference in interpretation of axes in MDS space is mainly because we did not include happy expressions in order to focus our investigation on negative emotions.

In the future, it is important to confirm that the observed patterns can be generalized to positive emotions. Alternatively, additional data such as valence of the facial expressions could be included in analyzing MDS to help in explaining what the axes might represent (Hollins et al., 1993, 2000). However, the main purpose of using MDS in our study is not to examine what the MDS space represents, but how tears affect distances between stimuli in the MDS space. Thus, while the interpretation of axes in MDS in our study is mainly speculative, it does not undermine our findings that supported the sadness enhancement hypothesis.

Third, we have asked Americans (with the majority of them self-identified as Caucasian Americans) to evaluate Asian facial expressions. This is based on the assumptions of cultural universalism of emotional facial expressions (Ekman et al., 1969) and the previous findings supporting the cultural universalism of the tears effect; tears increased sadness ratings in facial expressions in East Asian countries (Takahashi et al., 2015) and the United States (Provine et al., 2009). Actually, we conducted a preliminary study to ensure the selected facial expressions are categorized at a high accuracy rate (at least 80%) by both Caucasians Americans (*n* = 17) and Asian Americans (*n* = 3). However, because cultural differences can affect emotion ratings, it is important to further investigate the cross-cultural differences of the tears effect.

Fourth, the manipulation of tears was done so that tears were rolling down on both sides of the faces. This was done to ensure that the tears were visible to the participants and also to standardize the manipulation (instead of having tears on just one side of the faces). However, such tears could be uncommon and rarely observed in reality. It is also possible that such intense tears could have been associated with the perception of excessive or intense emotions. While the tears in our study were deliberately made to be intense, the intense tears did not lead to the perception of excessive emotions (i.e., did not support the general enhancement hypothesis), but showed an increase in sadness ratings for all facial expressions, supporting the sadness enhancement hypothesis. Although it is unlikely that more-ecologically valid tears favor the general enhancement hypothesis, future research could examine tearing of various intensity (i.e., more subtle or naturalistic) and its effect on emotion perception.

## Conclusion

Our study showed that tears provide an additional emotional signal on top of those by muscular contraction and relaxation within the face. Specifically, in the absence of additional contextual information, tears increased the intensity of perceived sadness and caused minor effects on the intensity of other negative basic emotions. The visualization of MDS spaces showed that facial expressions with tears were perceived as more similar to sad expressions. These results, in combination, support the sadness-enhancement hypothesis.

## Author’s Note

This research was conducted as a part of the second author’s Ph.D. dissertation.

## Ethics Statement

This study was carried out in accordance with the recommendations of Nanyang Technological University’s Institutional Review Board with informed consent from all subjects. All subjects gave online informed consent in accordance with the Declaration of Helsinki. The protocol was approved by the Nanyang Technological University’s Institutional Review Board.

## Author Contributions

KI and RK conceived the study. KI and CO designed the experiments. CO carried out the experiments and analyzed the data. KI, CO, and RK wrote the manuscript and approved the final version of the submitted manuscript.

## Conflict of Interest Statement

The authors declare that the research was conducted in the absence of any commercial or financial relationships that could be construed as a potential conflict of interest.

## Footnotes

1 The general enhancement hypothesis can take negative values when the ratings of non-primary emotions were over 1. However, as shown in the graph corresponding to the general hypothesis in Supplementary Figure S2, the chances of getting a value smaller than what we found in our results are low.
