# Supplementary material for: Emotional Tears Communicate Sadness but Not Excessive Emotions Without Other Contextual Knowledge
Source: Front Psychol. 2019 Apr 24;10:878. doi: 10.3389/fpsyg.2019.00878 (PMC6491854; doi:10.3389/fpsyg.2019.00878)
Supplement: Supplementary file 1 [file Data_Sheet_1.pdf]

Supplementary Material

Table S1
Hypotheses and expected dissimilarities

	Emotion rating				Dissimilarity from sad expression (1-r, in the main analysis)	Dissimilarity from sad expression (rating space, supplemental analysis)
	Anger	Disgust	Fear	Sadness		
Non-tearful facial expressions						
Facial Expressions						
Anger	5	1	1	1	1.33	5.66
Disgust	1	5	1	1	1.33	5.66
Fear	1	1	5	1	1.33	5.66
Sadness	1	1	1	5	0.00	0
Tearful expressions with sadness enhancement hypothesis (assuming different amounts of effects with Weber's law)						
Anger	4	1	1	5	0.27	3.61
Disgust	1	4	1	5	0.27	3.61
Fear	1	1	4	5	0.27	3.61
Sadness	1	1	1	7	0	0.00
Tearful expressions with sadness enhancement hypothesis (assuming the same effect on all facial expressions)						
Anger	5	1	1	5	0.42	5.66
Disgust	1	5	1	5	0.42	5.66
Fear	1	1	5	5	0.42	5.66
Sadness	1	1	1	9	0	0
Tearful expressions with general enhancement hypothesis (assuming the same effect on all facial expressions)						
Anger	9	1	1	1	1.33	11.31
Disgust	1	9	1	1	1.33	11.31
Fear	1	1	9	1	1.33	11.31
Sadness	1	1	1	9	0	0
Tearful expressions with general enhancement hypothesis (assuming greater effects on sad facial expressions)						
Anger	7	1	1	1	1.33	10.00
Disgust	1	7	1	1	1.33	10.00
Fear	1	1	7	1	1.33	10.00
Sadness	1	1	1	9	0	0

Note: The ratings in this table are hypothetical.

Table S2
Mean Euclidean Distances between Sad Expressions and Other Facial Expressions in MDS Space Combining Tearful and Non-Tearful Conditions of group mean data

	Tearful	Non-Tearful
Anger	0.94	1.09
Disgust	1.26	1.41
Fear	0.71	1.05
Neutral	0.46	0.71

Table S3
Mean Euclidean Distances between Sad Expressions and Other Facial Expressions in four raw emotion ratings

	Tearful	Non-Tearful
Anger	76.80 *(30.71)*	87.16 *(27.36)*
Disgust	85.63 *(31.90)*	98.30 *(30.63)*
Fear	70.30 *(25.21)*	86.80 *(29.52)*
Neutral	58.73 *(21.65)*	67.22 *(21.57)*

Note: Standard deviations are given in parentheses.

In order to confirm the results of the MDS analysis in the main text, we computed Euclidean distances between each sad expression and every other facial expression (i.e., angry, disgusted, fearful, and neutral) in four dimensions of emotion ratings, assuming they are orthogonal to each other. This approach is directly related to Figure S3 and complementary to the method used in the main text because this analysis uses a different dissimilarity measure (Euclidean distance in dimensions of raw emotion ratings vs. 1- correlation coefficient). Table S3 shows the mean and standard deviation of the distances between sad expressions and each of the other expressions.

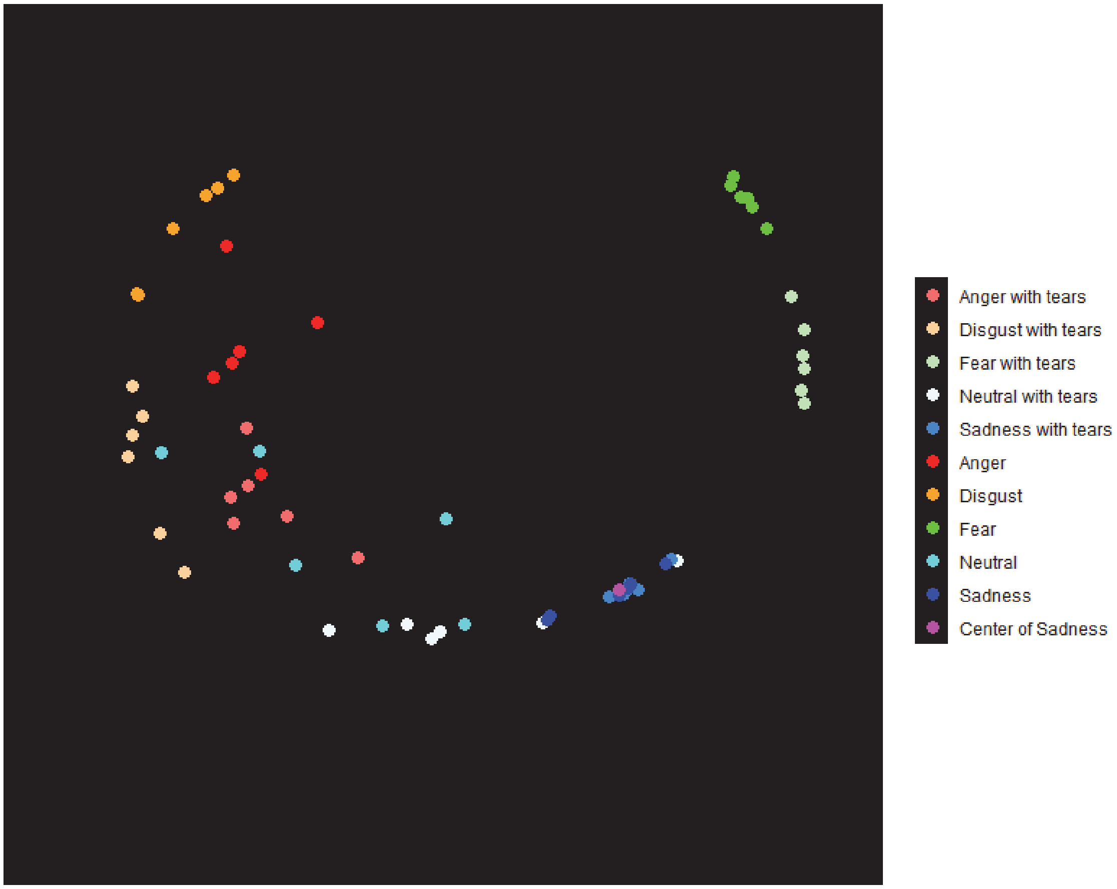

Figure S1. Multidimensional Scaling plot for comparing dissimilarities among tearful vs. non-tearful facial expressions.

In this analysis, we conducted the MDS analysis wherein stimuli with tears and without tears are mapped in the same space. Because we used a between-subject design, we conducted this analysis at the group level (i.e., group mean). The addition of tears reduces the distances between sad expressions and other facial expressions, supporting the sadness enhancement hypothesis. The means and standard deviations of distances between tearful and non-tearful condition were shown in Table S2.

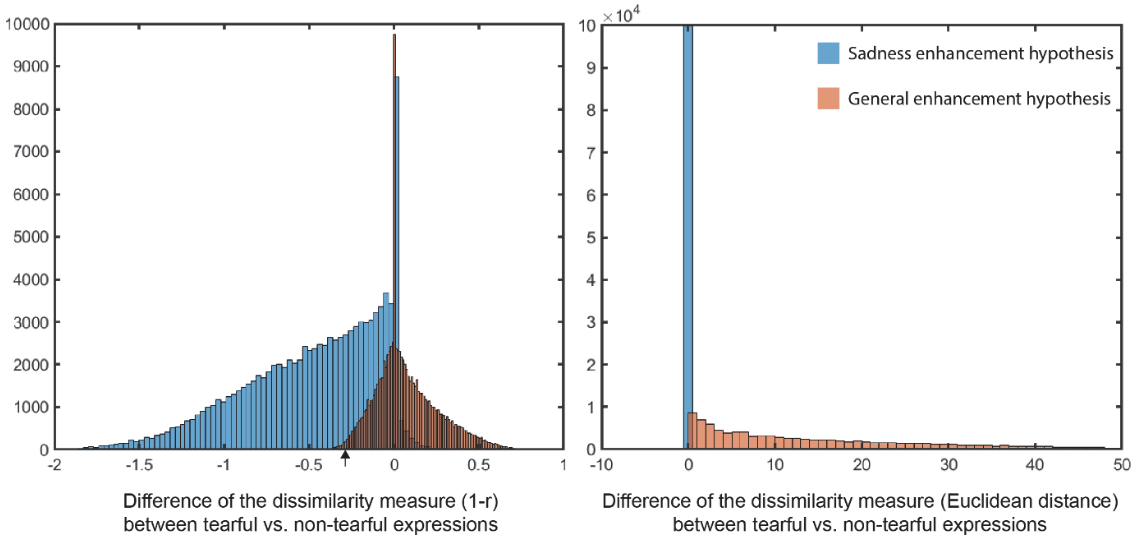

Figure S2. Histogram of simulated dissimilarities

We conducted 100,000 simulations on the differences in dissimilarity between sad expressions and other facial expressions for tearful and non-tearful conditions. The rating scale for emotion intensity takes integers from 0 to 99. We assigned randomized integers to the intensity ratings of two stimuli and controlled for several factors: (a) the ratings of the primary emotion of the expressions were set to be higher than the ratings of the other emotions, (b) the increments in ratings due to the tears effect were less than the ratings of primary emotion, and (c) the increments due to the tears effect were the same in both stimuli. The graph on the left shows the results of the dissimilarities obtained by 1 – r and the graph on the right shows the results of dissimilarities obtained by Euclidean distance in the supplemental analysis. The chances of observing the differences with a value smaller than what we found in this study (-0.28, an arrow in the left figure) was less than 5 % of the results of the simulation for the general enhancement hypothesis. Note that in the figure on the right, the sadness enhancement hypothesis produces only zero.

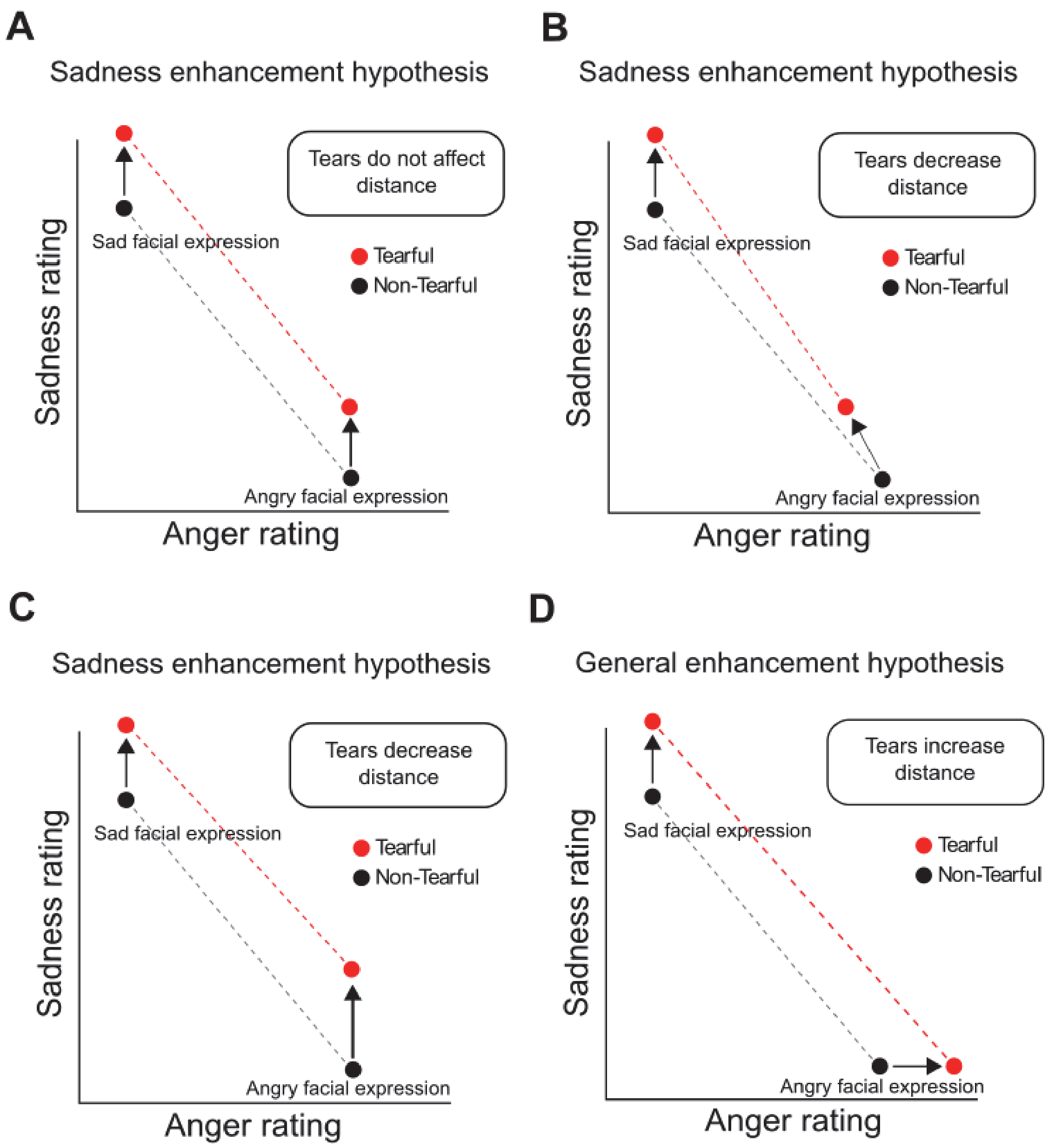

Figure S3. Sadness Enhancement Hypothesis and General Enhancement Hypothesis in Multidimensional Space of Emotions

The figure shows examples of how the hypotheses are associated with the space of emotion ratings. Before the transformation by MDS, stimuli were expressed in four dimensions (sadness, anger, fear, and disgust). This figure shows two dimensions (sadness and anger ratings) for simplicity. If the data supports the sadness enhancement hypothesis, tears would increase the sadness ratings in all emotional facial expressions. (A) If tears increase sadness ratings in all expressions similarly, then the distances between the stimuli would not change. (B) If tears increase sadness ratings in all expressions and reduce other ratings in non-sad expressions, the distances between the stimuli would decrease. (C) If tears lead to a smaller increment of sadness rating in sad facial expressions due to Weber's law (Takahashi et al., 2015; Gao et al., 2010, 2013), the distances between the stimuli would decrease. (D) By contrast, if the data supports the general enhancement hypothesis, tears would increase

sadness in sad expression and anger in angry expression. As a result, the distance between facial expressions would increase. Thus, changes in the distances from sad facial expressions can determine if data supports either sadness enhancement hypothesis or general enhancement hypothesis. In our main analysis, we used 1 – r (correlation coefficient) instead of Euclidean distances as dissimilarity measures and translated them in the MDS space. Nevertheless, the distances can be still used to test the hypotheses.
